# Efficacy and Safety of Different Dosing Regimens of Rivaroxaban in Patients With Atrial Fibrillation for Stroke Prevention: A Systematic Review and Meta-Analysis

**DOI:** 10.7759/cureus.51541

**Published:** 2024-01-02

**Authors:** Apurva Popat, Sagar K Patel, Susmitha Adusumilli, Ahmed Irshad, Aishwarya Nagaraj, Krisha K Patel, Stavan Y Jani, Gul Nawaz, Abdul Wahab, Satya Bora, Lakshay Mittal, Sweta Yadav

**Affiliations:** 1 Internal Medicine, Marshfield Clinic Health System, Marshfield, USA; 2 Internal Medicine, Gujarat Adani Institute of Medical Sciences, Bhuj, IND; 3 College of Medicine, Chongqing Medical University, Chongqing, CHN; 4 Internal Medicine, Faisalabad Medical University, Faisalabad, PAK; 5 Surgery and Pharmacology, Our Lady of Fatima University, Bangalore, IND; 6 College of Medicine, Dr. M. K. Shah Medical College and Research Center, Ahmedabad, IND; 7 Internal Medicine, Bukovinian State Medical University, Chernivtsi, UKR; 8 Internal Medicine, Allama Iqbal Medical College, Lahore, PAK; 9 Internal Medicine, Sargodha Medical College, Sargodha, PAK; 10 Neurology, Dr. Pinnamaneni Siddhartha Institute of Medical Sciences and Research Foundation, Vijayawada, IND; 11 Internal Medicine, Pandit Bhagwat Dayal Sharma Post Graduate Institute of Medical Sciences, Rohtak, IND; 12 Internal Medicine, Gujarat Medical Education and Research Society (GMERS) Medical College, Ahmedabad, IND

**Keywords:** real-world evidence, hemorrhagic stroke, clinical decision-making, personalized medicine, efficacy and safety, systematic review and meta-analysis, stroke prevention, anticoagulation therapy, rivaroxaban dosing, atrial fibrillation

## Abstract

Atrial fibrillation (AF) poses a substantial risk of stroke, necessitating effective anticoagulation therapy. This systematic review and meta-analysis (SRMA) evaluates the efficacy and safety of different dosing regimens of rivaroxaban in patients with AF. A comprehensive search of relevant databases, focusing on studies published from 2017 onward, was conducted. Inclusion criteria comprised randomized controlled trials (RCTs) and observational studies comparing standard and reduced dosing of rivaroxaban in AF. Data extraction and risk of bias (ROB) assessment were performed, and a meta-analysis was conducted for relevant outcomes. A total of 21 studies fulfilled the inclusion criteria. Standard dosing demonstrates a slightly lower risk of composite effectiveness outcomes and safety outcomes (HR: 0.79, 95% CI: 0.66-0.94, P=0.01) compared to reduced dosing (HR: 0.83, 95% CI: 0.71-0.97, P=0.02). Notable differences in major bleeding, gastrointestinal bleeding (GIB), and intracranial bleeding favored standard dosing. Hemorrhagic stroke and all-cause stroke rates differed significantly, with standard dosing showing a more favorable profile for ischemic stroke prevention. This study highlights the pivotal role of personalized anticoagulation therapy in AF. Standard dosing of rivaroxaban emerges as a preferred strategy for stroke prevention, balancing efficacy and safety. Clinical decision-making should consider individual patient characteristics and future research should delve into specific subpopulations and long-term outcomes to further refine treatment guidelines. The study bridges evidence from clinical trials to real-world practice, offering insights into the evolving landscape of AF management.

## Introduction and background

Atrial fibrillation (AF), characterized by irregular heartbeats, remains a critical public health concern due to its association with an elevated risk of stroke and systemic embolism [1]. Approximately three to six million individuals in the United States are currently affected by AF, and it is anticipated that these figures will increase to a range of six to 16 million by the year 2050 [2]. In addressing this risk, anticoagulation therapy has become integral, with direct oral anticoagulants (DOACs) emerging as pivotal agents. Rivaroxaban, a factor Xa inhibitor, stands out among DOACs, demonstrating efficacy in stroke prevention with a potentially more convenient once-a-day dosing regimen [3]. Recent advancements in the dosing strategies of rivaroxaban have garnered significant attention. The exploration of different daily dosing regimens has become a focal point, offering potential implications for patient adherence and outcomes [4]. Moreover, the comparative effectiveness of rivaroxaban with other anticoagulants, especially vitamin K antagonists (VKAs), remains a subject of ongoing scrutiny. Recent trials have aimed to elucidate the relative benefits and risks of rivaroxaban in comparison to traditional anticoagulants [5].

Rationale

The selection of an optimal anticoagulation strategy for stroke prevention in patients with AF is a critical clinical decision with direct implications for patient outcomes. Rivaroxaban, a DOAC, has emerged as a prominent therapeutic option, demonstrating efficacy and safety in large-scale trials [6]. However, the dosing regimen of rivaroxaban, either standard or reduced dose, presents a nuanced aspect that warrants a comprehensive evaluation. Several recent studies have investigated the impact of different dosing regimens of rivaroxaban on both efficacy and safety outcomes in patients with AF. Understanding the comparative effectiveness of standard versus reduced dosing is crucial for tailoring anticoagulation therapy to individual patient needs, optimizing adherence, and potentially improving clinical outcomes.

Objectives

The objectives of this analysis are (i) to assess and compare the efficacy of standard dosing of rivaroxaban (e.g., 20 mg once daily (OD)) with reduced dosing regimens (e.g., 15 mg OD) in preventing stroke and systemic embolism in patients with AF. (ii) Explore variations in thromboembolic events, including ischemic strokes, between standard and reduced dosing strategies. (iii) Evaluate and compare the safety profiles of standard and reduced dosing of rivaroxaban in patients with AF. (iv) Conduct a thorough assessment of publication bias by analyzing and reporting on potential selective reporting of outcomes in the included studies.

## Review

Definitions

Atrial Fibrillation

The abnormal cardiac rhythm is characterized by rapid, uncoordinated firing of electrical impulses in the upper chambers of the heart (heart atria). In such cases, blood cannot be effectively pumped into the lower chambers of the heart (heart ventricles). It is caused by abnormal impulse generation (Pubmed: MeSH).

Stroke Prevention

Stroke prevention in the context of AF involves measures to reduce the risk of thromboembolic events, particularly ischemic strokes. It encompasses anticoagulant therapy to mitigate the formation of blood clots in the atria [7].

Standard and Reduced Dosing

Standard dosing of rivaroxaban refers to the recommended dosage established by regulatory agencies and clinical guidelines for the prevention of stroke in patients with AF. For example, the standard dose might be 20 mg of rivaroxaban administered OD. Reduced dosing of rivaroxaban involves the use of a lower dosage than the standard recommendation and is designed to achieve a balance between efficacy and safety in specific patient populations. An example could be a reduced dose of 15 mg of rivaroxaban OD [8].

Efficacy and Safety

Efficacy refers to the ability of rivaroxaban to achieve its intended therapeutic effect, primarily the prevention of stroke and systemic embolism in patients with AF, while safety encompasses the assessment of adverse events associated with the use of rivaroxaban, including major bleeding events, clinically relevant non-major bleeding, and overall bleeding complications [9].

Methods

Eligibility Criteria

We set the eligibility criteria for studies following the Population, Intervention, Comparison, Outcome, and Study Design (PICOS) scheme, as recommended by Preferred Reporting Items for Systematic Review and Meta-Analysis (PRISMA).

The inclusion criteria were as follows: (1) Studies that were published between 2017 and 2023; (2) adults with a confirmed diagnosis of AF. (3) Studies investigating different dosing regimens of rivaroxaban. (4) Studies comparing different dosing regimens of rivaroxaban with other anticoagulants. (5) Studies reporting efficacy in stroke prevention and safety outcomes (bleeding events). (6) Studies with abstracts and/or free full-texts available were selected.

The exclusion criteria were: (1) Studies older than 2017; (2) study designs such as narrative reviews were not included in this study; (3) studies, especially RCTs (randomized control trials), with a “high” risk of bias (ROB) identified through Cochrane ROB calculator tool available online; (4) studies that included young pediatric population; (5) Studies that demonstrated wrong outcomes for our measured variables (discussed later).

Information Sources

We searched a number of digital databases for relevant literature. These include PubMed, Google Scholar, ClinicalTrials.gov, ScienceDirect, Medline, and Embase. Independent journals and other independent sources were also included. The “Journal of Thrombosis and Thrombolysis,” “JAMA Network,” “BMJ,” “Elsevier,” “American Heart Association (AHA) Journal,” and others were the sources of literature other than databases.

Search Strategy

We found a total of 21 studies (n=730) that were eligible for the inclusion criteria and covered the terms: ("atrial fibrillation" OR "AF") AND ("rivaroxaban" AND ("dosage" OR "dosing regimen" OR "once daily" OR "twice daily")) AND ("stroke prevention" OR "efficacy" OR "safety"), Filters: Abstract, Free full text, Clinical Study, Clinical Trial, Meta-Analysis, Randomized Controlled Trial, Systematic Review, in the last 5 years, Humans, English. Additionally, we inspected the reference lists of the studies selected for the systematic review and the meta-analysis.

Selection Process

A group of three researchers searched for literature in peer-reviewed journals and publications in accordance with the inclusion criteria. After a thorough selection of the literature, peer-reviewed journals with a strong impact factor were explored to reduce the risk of publication bias. All selected studies were uploaded to the screening software Rayyan.ai for primary and secondary screening of the literature [10]. Three researchers worked as collaborators to "include" or "exclude" eligible studies based on the inclusion and exclusion criteria. A total of 21 studies (n=291) were considered for the final review and analysis. Studies that did not pass the eligibility for screening were put under “exclusion” or “dispute.” We created a team of three researchers for study selection to serve as tiebreakers for a disputed study. Exclusion reasons were put forward before excluding a study from the literature. Studies were excluded because (1) there was a problem with the population; (2) the study design was not ideal for our analysis; (3) the study measured the wrong outcomes; or (4) we found a high ROB. Sometimes, it was a combined effect of multiple reasons for exclusion.

Data Items

**Figure 1:**
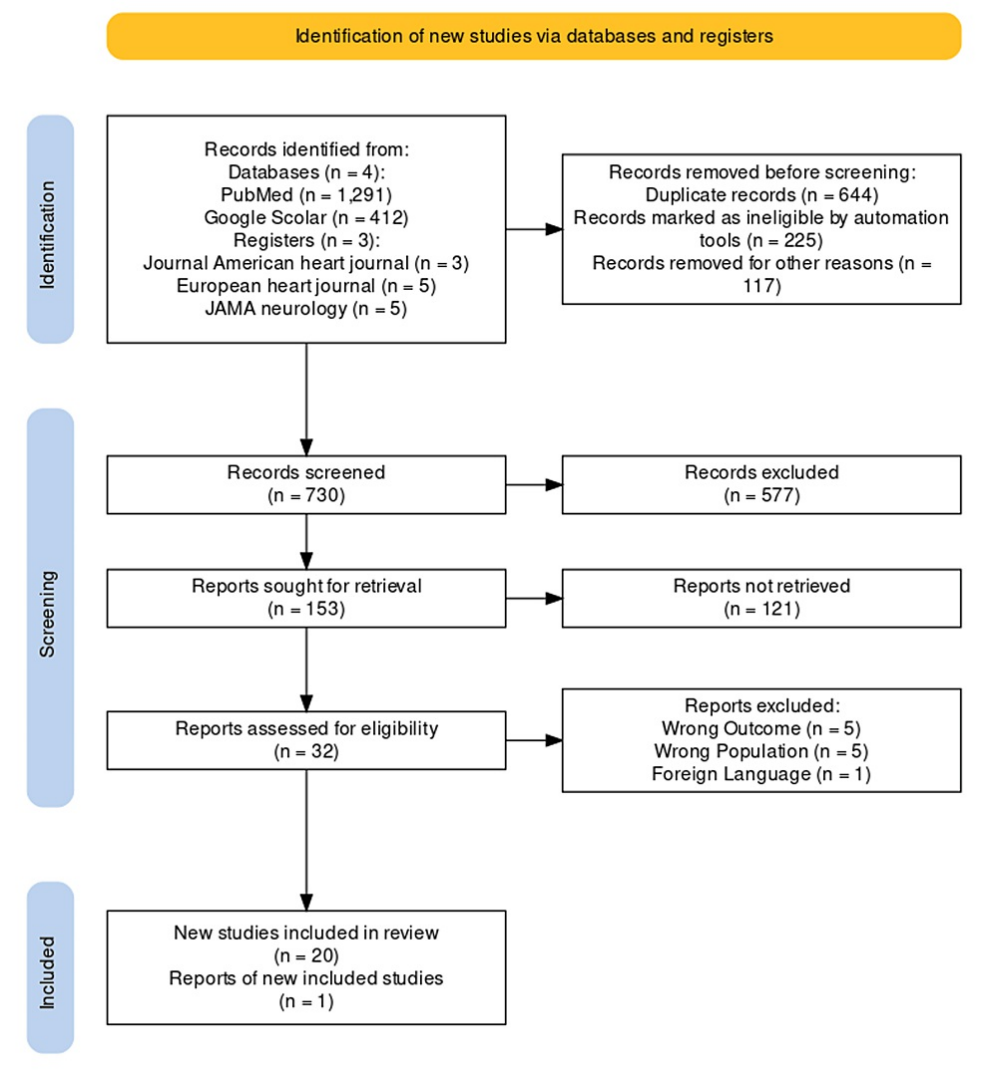
PRISMA flow chart for selected studies

The total sample size for the selected literature (n=21) was scrutinized after the secondary screening protocol was completed. We used the PRISMA standards to create a PRISMA flow diagram for the selected studies from journals and other independent resources (if the reports were available) [11]. The PRISMA flow diagram is given in Figure 1.

After the study selection process was complete, we tabulated the study interventions one by one against the study population and the outcomes studied. Only the relevant themes of the outcome were mentioned in the synthesis table.

Bias in the analysis was minimized by (1) selecting high-quality research and thorough literature review, (2) eliminating the double standard concerning peer review and informed consent applied to clinical research and practice, (3) requiring peer reviewers to acknowledge conflicts of interest, (4) replacing ordinary review articles with meta-analyses. Systematic reviews and narrative reviews were frequently excluded from the literature to maintain the standards of the study. These guidelines detect and remove bias in the study protocol in accordance with Chalmers et al. stages of removing publication bias [12]. All the studies chosen for the meta-analysis were found to have a “low” overall ROB. A “traffic light” figure was plotted using this data for randomization. A summary of the ROB was also mentioned for collaborator convenience.

Quality Assessment

For systematic review

All the studies selected for quality assessment were analyzed for publication bias. All the studies were manually checked for intervention characteristics, population demographics, and outcomes domains. All the studies selected for meta-analysis underwent quality assessment using the Critical Appraisal Skill Program (CASP) tool. The quality assessment included three broad categories of questions: (1) were the study results validated? (2) what were the results? (3) are the results of the study applicable locally? 11 questions for quality assessment were answered with careful consideration of study designs and the relevant outcomes. The questions were answered in terms of “Yes,” “No,” and “Can’t tell.” If the answer to the first question is “yes,” it is worth proceeding with the remaining questions. There is some degree of overlap between the questions. The description of the answers and researcher remarks has also been mentioned in Table 1.

**Table 1:** Quality assessment using CASP tool KEY: Y=YES, N=NO, ?=cannot tell CASP, Critical Appraisal Skill Program; CI, confidence interval

S. no.	Questions	Ray et al. [15]	Lau et al. [16]	Alberts et al. [17]	Huang et al. [18]	Jansson et al. [19]
1	Did the study address a clearly focused issue?	?	Y	Y	Y	Y
2	Did the authors use an appropriate method to answer their question?	Y	?	Y	Y	Y
3	Were the cases recruited in an acceptable way?	?	Y	Y	Y	Y
4	Were the controls selected in an acceptable way?	Y	Y	Y	Y	Y
5	Was the exposure accurately measured to minimize bias?	N	N	Y	Y	?
6(a)	Aside from the experimental intervention, were the groups treated equally?	Y	Y	N	Y	Y
6(b)	Have the authors taken account of the potential confounding factors in the design and/or in their analysis?	N	Y	?	?	Y
7	How large was the treatment effect?	The study measures HR: 1.12; 95% CI: 1.05–1.21, P=0.01.	HR: 0.72; 95% CI: 0.66–0.79, P=0.01	The study predicted close HR values for outcome. HR: 0.81; 95% CI: 0.73–0.91, P=0.01	HR: 0.79; 95% CI: 0.66–0.94, P=0.01	The study showed a significant Standardized absolute risk: 1.73% (1.43% to 2.03%)
8	How precise was the estimate of the treatment effect?	p<0.0001. The results validate the study hypothesis.	P=0.05. The overall effect size showed no significance	The analysis had a linear relation (p=0.05)	Statistically significant association with p<0.001	Statistically significant association with p<0.001
9	Do you believe the results?	Y	Y	Y	Y	?
10	Can the results be applied to the local population?	Y	N	Y	N	N
11	Do the results of this study fit with other available evidence?	N	Y	?	Y	?
Score out of 11		7	9	9	10	8

For meta-analysis

We sought digital/online tools for risk-of-bias assessment of the studies selected for the meta-analysis. All the studies, except RCTs, were assessed by an online tool via CASP to create a quality assessment table for all the studies included in the meta-analysis. The assessment table for five studies is shown in Table 1. Further all the primary studies, that is, RCTs eligible for the analysis were independently selected based on the Cochrane criteria for ROB. We calculated the ROB via the Cochrane Risk-of-Bias (version 2019) online tool [13]. Cochrane Risk-of-Bias (version 3.5.1) online tool was used to assess seven domains of risk occurring in the primary studies (ROBv2 tool). The risk-of-bias domains that were analyzed for the meta-analysis were as follows: (1) random sequence generation (selection bias), (2) allocation concealment (selection bias), (3) blinding of participants and personnel (performance bias), (4) blinding of outcome assessment (detection bias), (5) incomplete outcome data (attrition bias), (6) selective reporting (reporting bias), and (7) other bias [13]. Continuous data was extracted for the statistical meta from eight out of 21 primary studies. We created a “forest plot” using Review Manager (RevMan version 5.4) for the meta-analysis. Meta-analysis of eight primary studies (study design= RCTs) was done using Revman (version 3.5.1). Three researchers collected comparable and poolable data for the analytical tool [14]. All the data was available in the form of continuous variables. The data for the meta-analysis is provided in the results section of our study.

Results

Study Characteristics

The final sample for the systematic analysis included 21 peer-reviewed studies, 13 RCTs, and seven cohort studies. Seventeen of these studies used randomization, and 11 used a quasi-experimental design, eight of which used Cox regression methods to construct a matched comparison group. One study used latent curve modeling as well. Sample sizes ranged from as small as N=175 to as large as N=227,572. Follow-up data collection time points ranged from two months to 24 months (two years). The results of the systematic review revealed a total of 17/21 (77%) studies advocating the effectiveness of standard dosing of rivaroxaban. On the other hand, 5/21 (23%) studies concluded “no effect” or “negative” association of standard dosing compared to reduced one. The synthesis table for the systematic review is given in Table 2.

**Table 2:** Comparative analysis of rivaroxaban dosing in various clinical trials and studies RCT, randomized control trial; DOACs, direct oral anticoagulants; NOACs, non-vitamin K antagonist oral anticoagulants; ICH, intracranial hemorrhage; GIB, gastrointestinal bleeding; AF, atrial fibrillation; MHV, mechanical heart valve; HFrEF, heart failure with reduced ejection fraction; ESUS, embolic stroke of undetermined source; ICD-9-CM, Classification of Diseases, Ninth Revision, and Clinical Modification; NVAF, non-valvular atrial fibrillation; RHD, rheumatic heart disease; MVA, mitral valve area; DTIs, direct thrombin inhibitors; FXaIs, factor Xa inhibitors; INR, international normalized ratio; CrCl, creatinine clearance; BW, body weight; VKA, vitamin K antagonist; RR, relative risk; CI, confidence interval; HR, hazard ratios; TTR, time in therapeutic range; SE, systemic embolism; MI, myocardial infarction; PES, paroxysmal embolic strokes; SS, silent stroke; IG, intervention group; UG, control group; TIA, transient ischemic attack; ISTH, International Society on Thrombosis and Hemostasis

Sr. no	Study ID	Location	Study design	Participants	Intervention	Main findings
1	Ray et al. [15]	USA	Retrospective cohort study	581,451 of US citizens 65 years or older and younger persons with disabilities, provided the study data.	Apixaban or rivaroxaban with either the standard (5 mg twice daily for apixaban and 20 mg OD for rivaroxaban) or reduced (2.5 mg twice daily for apixaban and 15 mg OD for rivaroxaban).	The use of rivaroxaban, when contrasted with apixaban, was linked to a notable rise in the likelihood of experiencing major ischemic or hemorrhagic events. Moreover, the incidence of nonfatal extracranial bleeding in the rivaroxaban cohort was higher compared to the apixaban cohort. Individuals who received reduced doses exhibited a higher prevalence of additional risk factors for stroke, as indicated by a higher mean CHA2DS2-VASc score (5.0 vs 4.1) and an increased risk of bleeding.
2	Lau et al. [16]	France, Germany, UK, USA	Multinational population-based cohort study	527,226 individuals who initiated treatment with DOACs. These included 281,320 users of apixaban, 61,008 users of dabigatran, 12,722 users of edoxaban, and 172,176 users of rivaroxaban.	Apixaban, dabigatran, endoxaban, or rivaroxaban with standard and reduced dose.	In individuals with AF, the use of apixaban was linked to a reduced risk of GIB and comparable rates of ischemic stroke or SE, ICH, and all-cause mortality when compared to dabigatran, edoxaban, and rivaroxaban. This observation held true for both patients aged 80 years or older and those with CKD, demographics that are frequently underrepresented in clinical trials.
3	Alberts et al. [17]	USA	Retrospective cohort study	20,473 de-identified patients from the Optum Clinformatics Database. Rivaroxaban, n=6876 and warfarin, n=13,597	Treatment with rivaroxaban or warfarin within 30 days following initial diagnosis of NVAF.	Individuals receiving rivaroxaban, as opposed to warfarin, experienced a notable decrease in the risk of stroke, particularly severe strokes, as well as a reduction in all-cause mortality following a stroke.
4	Huang et al. [18]	Taiwan	Retrospective cohort study	The inclusion criteria were met by 24,101 individuals aged 20 or older, who had at least one inpatient or two distinct outpatient diagnoses of AF determined by the International ICD-9-CM code 427.31. These individuals were prescribed either rivaroxaban or warfarin between June 1, 2012, and December 31, 2015.	Rivaroxaban (20mg, 15mg, 10mg) or warfarin.	Rivaroxaban demonstrated a significantly reduced risk for both composite effectiveness outcomes (HR: 0.79; 95% CI: 0.66–0.94, P=0.01) and safety outcomes (HR 0.83; 95% CI: 0.71–0.97, P=0.02) when compared to warfarin.
5	Jansson et al. [19]	Sweden	Registry-based retrospective cohort study	After excluding 374,135 patients who were not warfarin or DOAC naïve were not prescribed a reduced dose, had a previous MHV, or were under 18 years old, the study included 40,564 patients. Among them, 11,083 received newly initiated DOAC treatment (apixaban, dabigatran, or rivaroxaban), while 29,481 patients were treated with warfarin.	Included in the study were patients with NVAF who were enrolled in the Auricula registry and were prescribed a new treatment regimen involving apixaban, dabigatran, rivaroxaban, or warfarin. The study specifically focused on patients who were treated with reduced doses of DOACs, such as apixaban 2.5 mg twice daily (BD), dabigatran 110 mg twice daily (BD), or rivaroxaban 15 mg OD.	There were notable variations in the rates of major bleeding, GIB, and intracranial bleeding between the combined group of reduced-dose DOACs and warfarin treatment, with HR of 0.85 (95% CI 0.78–0.93), 0.81 (0.69–0.96), and 0.64 (0.51–0.80), respectively. Additionally, the rates of hemorrhagic stroke and all-cause stroke exhibited significant differences, with HR values of 0.68 (0.50–0.92) and 0.87 (0.76–0.99), respectively. A comparison between treatment with reduced-dose DOACs and high TTR warfarin demonstrated a clinically significant and favorable effectiveness and safety profile for reduced-dose DOACs. When comparing rivaroxaban with warfarin, rivaroxaban was associated with more effective prevention of ischemic stroke but a higher risk of major bleeding.
6	Carnicelli et al. [20]	Canada, Argentina, Taiwan, Scotland, Switzerland, USA, Germany, Japan	RCT	A total of 71, 683 patients were included (29,362 on standard-dose DOAC, 13,049 on lower-dose DOAC, and 29,272 on warfarin).	The four key trials comparing DOACs with warfarin in AF include RE-LY (Randomized Evaluation of Long-Term Anticoagulation Therapy), ROCKET AF (Rivaroxaban OD Oral Direct Factor Xa Inhibition Compared With Vitamin K Antagonism for Prevention of Stroke and Embolism Trial in Atrial Fibrillation), ARISTOTLE (Apixaban for Reduction in Stroke and Other Thromboembolic Events in Atrial Fibrillation), and ENGAGE AF-TIMI 48 (Effective Anticoagulation With Factor Xa Next Generation in Atrial Fibrillation–Thrombolysis in Myocardial Infarction 48).	When compared to warfarin, standard DOACs showed a markedly reduced risk of stroke or SE, death, and ICH. In contrast, lower-dose DOACs were not statistically different in terms of the risk of stroke, SE, or any other major hemorrhage. Consequently, among patients with AF, DOACs, particularly rivaroxaban, exhibit more favorable efficacy and safety profiles compared to warfarin.
7	Berwanger et al. [21]	Brazil	RCT	A total of 1005 individuals, aged 18 years or older, with permanent, paroxysmal, or persistent AF or flutter, and possessing a bioprosthetic mitral valve, were either receiving or intending to receive oral anticoagulation for the purpose of thromboembolism prophylaxis.	Evaluating patients with AF and a bioprosthetic mitral valve, the study compared the effects of rivaroxaban at a daily dose of 20 mg with dose-adjusted warfarin targeting an INR between 2.0 and 3.0.	Rivaroxaban demonstrated noninferiority to warfarin in terms of the average time until the primary outcome, defined as death, major cardiovascular events, or major bleeding at the 12-month mark.
8	Perera et al. [22]	Canada	RCT	Those eligible for the study included patients with stable atherosclerotic vascular disease. A total of 27,395 participants were randomized and monitored until February 6, 2017.	Participants received rivaroxaban (2.5 mg twice a day) plus aspirin (100 mg once a day), rivaroxaban (5 mg twice a day), or aspirin (100 mg once a day)	In individuals with systemic atherosclerosis, the combination of low-dose rivaroxaban and aspirin demonstrated substantial and statistically significant reductions in cardioembolic strokes and embolic strokes of undetermined source. Nevertheless, these findings from exploratory analysis should be independently validated before influencing clinical practices.
9	Blumer et al. [23]	Multi-national	RCT	Among the 14,264 patients subjected to randomization in the ROCKET AF trial, 1,878 individuals (13.2%) hailed from Latin America and were considered in this subgroup analysis. This Latin American subgroup comprised 569 patients from Argentina, 483 from Brazil, 286 from Chile, 268 from Colombia, 168 from Mexico, 84 from Peru, and 20 from Venezuela.	Individuals participating in the ROCKET AF trial were randomly allocated to receive either a fixed dose of rivaroxaban (20 mg OD or 15 mg OD for those with CrCl of 30-49 mL/min) or adjusted-dose warfarin (target INR 2.0-3.0). Additionally, patients in both groups were provided with placebo tablets to maintain the blinding of the study.	In Latin America, individuals with AF experienced comparable incidences of stroke and/or SE, elevated rates of vascular-related mortality, and reduced rates of bleeding when contrasted with patients from other global regions. The impact of rivaroxaban in comparison to warfarin in Latin America mirrored that observed in the rest of the world.
10	Akao et al. [24]	Japan	Randomized, multicenter, open-label, parallel-group trial	Men and women aged ≥20 years diagnosed with AF and stable CAD, patients with a baseline CHADS2 score ≥1	Participants were randomly allocated in equal proportions to one of two groups: the first receiving sole treatment with rivaroxaban (10 mg OD for individuals with a CrCl of 15–49 mL/min or 15 mg OD for those with a CrCl ≥50 mL/min), or the second undergoing combination therapy involving rivaroxaban and an antiplatelet agent (either aspirin or a P2Y12 inhibitor).	The use of rivaroxaban as a single treatment significantly lowered the main measures of both effectiveness and safety, and there were no indications of varying effects based on stroke risk. Additionally, there was no statistically significant variability observed across different patient risk categories for various endpoints, including stroke or SE, ischemic stroke, hemorrhagic stroke, MI or unstable angina, death from any cause, any bleeding, or overall adverse clinical events.
11	Mehra et al. [25]	Germany, Singapore, UK, USA, Netherlands	Double-blind, randomized trial	COMMANDER HF was an international, multicenter, double-blind, randomized clinical trial designed to assess the safety and effectiveness of rivaroxaban in comparison to a placebo in individuals with chronic HFrEF.	2,507 patients were randomly assigned to rivaroxaban and 2,515 to placebo.	Rivaroxaban at a dose of 2.5 mg BID reduced rates of stroke or TIA compared with placebo in this population.
12	Healey et al. [26]	Canada, Rome, Australia, Mexico, USA, Japan, China, Spain	RCT	The NAVIGATE ESUS trial involved the enrollment of 7,213 patients who had recently suffered an ischemic stroke and met the criteria for ESUS, which specifically refers to a nonlacunar stroke as confirmed by brain imaging.	Patients were randomly assigned, in a blinded fashion, to receive rivaroxaban (15 mg daily) or aspirin (100 mg daily).	Rivaroxaban demonstrated a decreased risk of recurrent stroke in individuals with ESUS and notable left atrial enlargement. However, it is essential for these findings to be validated independently before impacting clinical protocols. Notably, a daily dosage of 15 mg of rivaroxaban did not show a reduction in stroke risk compared to aspirin in ESUS patients. In contrast, it markedly diminished the risk of stroke in individuals with AF.
13	Zhang et al. [27]	45 countries	Multicenter, randomized, double-blind, double-dummy, event-driven trial	14,264 patients with NVAF, as documented on electrocardiography, who were at moderate-to-high risk of stroke.	In the ROCKET AF trial, individuals with NVAF were randomly assigned to receive either rivaroxaban (20 mg OD, or 15 mg OD if CrCl was 30-49 mL/min) or dose-adjusted warfarin (with a target INR of 2.0–3.0). The median follow-up duration for participants in this trial was 707 days.	The model-predicted rivaroxaban trough plasma concentration (C(trough)) did not show a significant association with efficacy outcomes. However, efficacy outcomes were found to be significantly associated with CrCl and a history of stroke. The relationship between exposure and major or non-major clinically relevant bleeding was shallow, with no evident threshold indicating an acceleration in risk. Notably, a history of GIB exerted a more pronounced influence on safety outcomes than C(trough). These findings provide support for the use of fixed rivaroxaban dosages of 15 mg and 20 mg OD in NVAF.
14	Shrestha et al. [28]	USA	Retrospective, observational cohort study	Patients with NVAF who were adults and had at least one DOAC pharmacy claim maintained continuous enrollment for at least 12 months following the initial DOAC claim and had a documented CrCl within three months prior to the index date were considered eligible. This information was sourced from the Optum/Humedica SmartFile database.	For apixaban, the prescribed dosage was either 5 mg or 2.5 mg twice daily. Dabigatran was administered at a dose of 150 mg twice daily for individuals with a CrCl of ≥30 mL/min, while those with a CrCl below 30 mL/min received a reduced dosage of 75 mg twice daily. Rivaroxaban was prescribed at a dosage of 20 mg OD for individuals with a CrCl greater than 50 mL/min, and a reduced dosage of 15 mg OD for those with a CrCl of 50 mL/min or lower.	Out of the 388 eligible patients, 69 individuals (17.8%) received inappropriate dosages, with rivaroxaban exhibiting the highest rate of inappropriate dosing. The majority of inappropriately dosed patients were underdosed. Notably, inappropriate dosing was identified in patients with both normal and insufficient renal function. It is emphasized that considering clinical factors beyond renal function is crucial to mitigate the risk of bleeding associated with DOAC therapy. While no significant difference in stroke risk was observed, it is important to note that very few stroke events were recorded in the study.
15	Stærk et al. [29]	Denmark	Retrospective cohort study	Patients were included if they had an AF diagnosis and subsequently filled a first-time prescription of OAC.	Dabigatran standard dose (150 mg, n=7,078), dabigatran reduced dose (110 mg, n=4,414), rivaroxaban standard dose (20 mg, n=6,868), rivaroxaban reduced dose (15 mg, n=2,098), apixaban standard dose (5 mg, n=7,203), and apixaban reduced dose (2.5 mg, n=3,861).	There were no significant differences in the risk of associated stroke/thromboembolism between standard and reduced doses of NOACs. However, rivaroxaban was linked to a higher risk of bleeding compared to dabigatran and apixaban. Conversely, dabigatran demonstrated a lower risk of intracranial bleeding compared to rivaroxaban and apixaban.
16	Pisters et al. [30]	Netherland	Prospective, observational study	6,784 patients who were diagnosed with non-valvular AF, started rivaroxaban therapy and provided written informed consent.	Rivaroxaban 20 mg OD, 15 mg daily, and 10 mg daily.	In routine clinical practice, patients receiving rivaroxaban exhibited low rates of major bleeding, instances of dosing that deviated from the labeled recommendations, and demonstrated high persistence rates over a one-year follow-up period.
17	Ntaios et al. [31]	Multi-national	RCT	7,213 patients were enrolled in the NAVIGATE-ESUS trial (38% women, mean age 67 years) and followed for a median of 11 months.	The NAVIGATE ESUS trial was a phase III study conducted internationally, employing a double-blinded, randomized design. It compared the effectiveness of rivaroxaban at a dose of 15 mg OD with aspirin at a dose of 100 mg OD in patients who had experienced a recent ESUS.	A significant number of patients with ESUS had multiple PES, and this factor could potentially account for the neutral findings in the NAVIGATE-ESUS trial. The recurrence rates between patients assigned to rivaroxaban and those assigned to aspirin were comparable across the range of PES.
18	Nagao et al. [32]	Japan	RCT	200 patients with non-valvular AF who were prescribed once-a-day DOACs including rivaroxaban and edoxaban, or twice-a-day DOACs including apixaban at the Chubu Rosai Hospital between April 2015 and January 2018.	The patients were categorized into two groups based on their dosing regimen of DOACs: interrupted or non-interrupted doses. Once-daily DOACs, such as rivaroxaban and edoxaban, were administered in one group, while the other group received twice-daily DOACs, specifically apixaban. For patients with mild renal dysfunction CrCl (30–50 mL/min), a lower dose of rivaroxaban (10 mg OD) was prescribed. The apixaban dose was determined based on factors such as age, BW, and renal function.	The incidence of SS in the IG was notably higher compared to the UG. Furthermore, the occurrences of symptomatic ischemic stroke/TIA or SS were similar among patients taking once-daily DOACs and those taking twice-daily DOACs in the IG. In the IG, the occurrence of SS was associated with intraoperative cardioversion and the duration of the procedure during AF ablation. This association might be supported by the observed differences in periprocedural PF1 + 2 value trends between the two groups.
19	Guimarães et al. [33]	Brazil	RCT	The eligible participants for the study were individuals aged 18 years or older, diagnosed with paroxysmal, permanent, or persistent AF or flutter, and had a bioprosthetic mitral valve with either planned or ongoing use of oral anticoagulation for the prevention of thromboembolism.	Individuals with a bioprosthetic mitral valve and AF or flutter were randomly allocated in a 1:1 ratio to receive either rivaroxaban at a daily dose of 20 mg (adjusted to 15 mg for those with CrCl) or dose-adjusted warfarin with a target INR of 2.0–3.0. The follow-up period for the study spanned 12 months.	The RIVER trial stands as the most extensive randomized trial to date that has been explicitly structured to evaluate the effectiveness and safety of a DOAC in individuals with bioprosthetic mitral valves and concurrent AF or flutter.
20	Karthikeyan et al. [34]	Multi-national	RCT	Eligible participants for the study were individuals aged 18 years and older with echocardiographically confirmed RHD and either current or past AF or atrial flutter. Additionally, patients were required to have a higher risk of stroke, determined by the presence of at least one of the following criteria: a CHA2DS2VASc score of ≥2, mitral stenosis with MVA ≤2 cm², or the presence of left atrial spontaneous echo contrast or thrombus.	Patients were randomly assigned to receive either rivaroxaban or VKA in a 1:1 ratio, utilizing a central, web-based randomization system. Those allocated to rivaroxaban were administered 20 mg of the drug once a day. However, patients with a CrCl below 50 mL/min received a reduced dose of 15 mg of rivaroxaban daily. Patients assigned to VKA were provided with any locally approved VKAs, with dosage adjustments made to maintain an INR within the range of 2-3.	The trial's primary efficacy outcome is a composite of stroke or SE. Secondary efficacy outcomes include the incidence of MI or vascular death. The primary safety outcome is major bleeding, as defined by the ISTH criteria. Additionally, a secondary safety outcome is the time taken for the occurrence of life-threatening or clinically relevant, non-major bleeding.
21	Providência et al. [35]	N/A	Meta-analysis	Phase III RCTs that investigated the efficacy and safety of DTIs and FXaIs compared to warfarin in patients with AF were considered eligible for inclusion.	In comparing treatment A (DTI) to B (warfarin) and treatment C (FXaI) to B (warfarin), the RR of treatment A (DTI) versus treatment C (FXaI) was estimated by utilizing a common comparator. Additionally, the comparison extended to different dosing regimens, specifically once-daily versus twice-daily dosing of NOACs.	In individuals with AF, NOACs exhibit an overall positive impact when compared to warfarin concerning the risk of stroke or SE, major bleeding, total and cardiovascular mortality, as well as intracranial bleeding. However, when the analysis is narrowed down to the two distinct pharmacologic classes, DTIs and FXaIs, as well as different dosing regimens such as once-daily and twice-daily NOACs, there is significant heterogeneity among studies, and no clear preference is observed in favor of any specific class or dosing regimen.

Risk of Bias Plot

**Figure 2:**
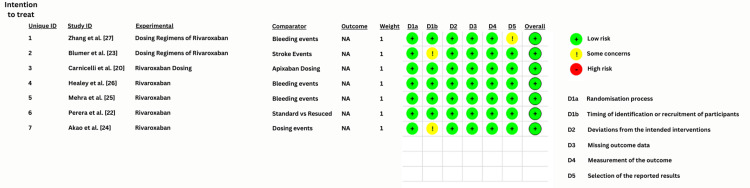
Cochrane ROB traffic plot ROB, risk of bias

As mentioned earlier, ROBv2 was used to assess the risk for all the primary studies selected for meta-analysis. The studies with a “low” overall ROB were then selected. We used the Cochrane Risk-of-Bias tool to create a “traffic lights” plot for the final assessment. The traffic plot for four studies is given below in Figure 2.

Forest Plot (Standard Dose)

**Figure 3:**
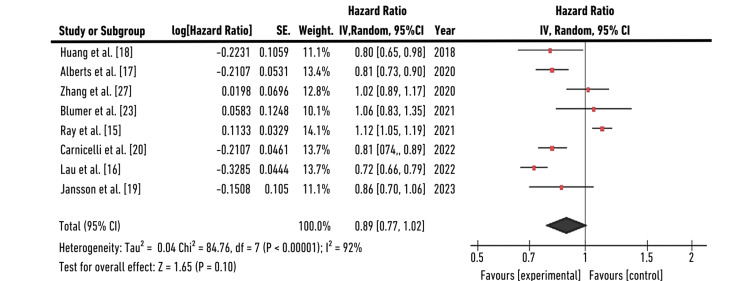
Forest plot for standard dose of rivaroxaban

Forest plot for eight individual studies was plotted for generalized inverse variance measuring hazard ratio (HR) as the primary outcome. A random-effects model was selected to calculate HR in terms of “log[HR]” and Standard Error “(SE)”. We calculated the confidence interval (CI=95%) on the horizontal axis, while the "point estimation" was represented by green squares on the plot. The total sample size (n=10609,175,161,2060,227572,29362,172176,2329) did not change significantly in the control groups. The central vertical line refers to a state of “no effect.” This forest plot summarized quantitative data about each study and provided an estimated overall quantitative value for all the combined effects. The overall combined effect size was calculated, which was found to be z=1.65, CI=95% (0.77, 1.02). The individual effect size was found to be significant for five out of eight studies: Huang et al., Alberts et al., Carnicelli et al., Lau et al., and Jansson et al. [17-20]. The heterogeneity was calculated to be Tau^2^=0.04; Chi^2^=84.76, df=7 (p-value=0.001); I^2^=92%. The analysis for the overall effect was found to be Z=1.65 (p=0.10). The individual effect of all studies favored the experimental group, that is, the population receiving the standard dose (SD=20 mg OD) of rivaroxaban. HR with a CI of 95% was found to be 0.80 (0.65,0.98) for Huang et al., 0.81(0.73,0.90) for Alberts et al., 1.02(0.89,1.17) for Zhang et al., 1.06(0.83,1.35) for Blumer et al., 1.12(1.05,1.19) for Ray et al., 0.81(0.74, 0.89) for Carnicelli et al., 0.72(0.66,0.79) for Lau et al., and 0.86(0.76,1.06) for Jansson et al. that indicates individual effects of five out of eight studies favored the experimental group, that is, the population receiving standard dose of rivaroxaban (20 mg) [15-20,23,27]. Use of rivaroxaban at doses of 20 mg and 15 mg was associated with a significantly lower risk of ischemic stroke (20 mg, HR: 0.48; 95% CI: 0.29-0.80, P=0.005; 15 mg, HR: 0.69; 95% CI: 0.53-0.90, P=0.005) and intracranial hemorrhage (ICH) (20 mg, HR: 0.24; 95% CI: 0.07-0.84, P=0.03; 15 mg, HR: 0.36; 95% CI: 0.21-0.62, P<0.001). The risk of the primary outcome was increased for rivaroxaban in both those receiving the reduced dose (RD, 6.4 (95% CI, 4.1-8.7); HR, 1.28 (95% CI, 1.16-1.40)) and the standard dose (SD, 1.8 (95% CI, 1.0-2.6); HR, 1.13 (95% CI, 1.06-1.21)) groups. Treatment with 20 mg rivaroxaban was associated with a lower risk of GIB (HR: 0.47; 95% CI: 0.24-0.90, P=0.02). The results for this study (HR=0.89, CI (0.77, 1.02)) were found to favor “standard dose therapy,” interpreting that efficacy and safety are non-significant with standard or reduced dosing of rivaroxaban. This study conformed to the analysis laid down by another meta-analysis by Providência et al. [35]. The forest plot for the meta-analysis is given in Figure 3. 

Forest Plot (Reduced Dose)

**Figure 4:**
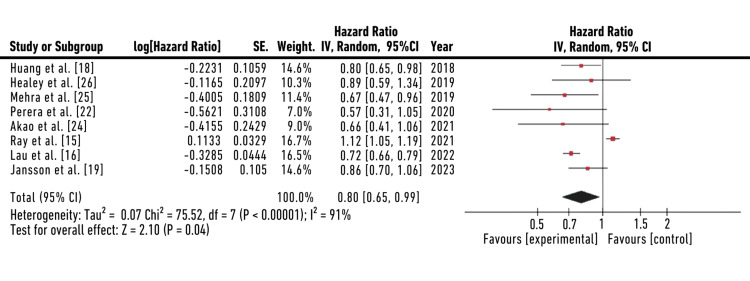
Forest plot for a reduced dose of rivaroxaban

The forest plot for reduced dosing of rivaroxaban summarized quantitative data about each study and provided an estimated overall quantitative value for all the combined effects. The overall combined effect size was calculated, which was found to be z=2.10, CI=95% (0.65, 0.99). The individual effect size was found to be significant for seven out of eight studies: Huang et al., Healey et al., Mehra et al., Perera et al., Akao et al., Lau et al., and Jansson et al. [16,18,19,22,24-26]. The heterogeneity was calculated to be Tau^2^=0.07; Chi^2^=75.52, df=7 (p-value=0.001); I^2^=91%. The analysis for the overall effect was found to be Z=2.01 (p=0.10). The individual effect of all studies favored the experimental group, that is, the population receiving a reduced dose (RD=15 mg OD) of rivaroxaban. HR with a CI of 95% was found to be 0.80 (0.65,0.98) for Huang et al, 0.89(0.59,1.34) for Healey et al., 0.67(0.47,0.96) for Mehra et al., 0.57(0.31,1.05) for Perera et al., 0.66(0.41,1.06) for Akao et al., 1.12(1.05,1.19) for Ray et al., 0.72(0.66,0.79) for Lau et al., and 0.86(0.76,1.06) for Jansson et al. that indicates individual effects of seven out of eight studies favored the experimental group, that is, the population receiving reduced dose of rivaroxaban (15 mg) [15,16,18,19,22,24-26]. The forest plot for a reduced dose of rivaroxaban is shown in Figure 4.

Discussion

The SRMA aimed to provide a comprehensive evaluation of the available evidence regarding the diverse dosing strategies of rivaroxaban in the context of AF. AF, a common cardiac arrhythmia, poses a significant risk of stroke, and anticoagulation therapy plays a pivotal role in its management. The choice between standard and reduced dosing regimens of rivaroxaban introduces a crucial decision point for clinicians, necessitating a thorough understanding of the associated efficacy and safety outcomes. Miyazaki et al.'s emphasis on the risks associated with under-dosing in anticoagulation therapy echoes the critical need for accurate dosing strategies, a key aspect highlighted in our study [36]. The efficacy and safety of different dosing regimens of rivaroxaban in patients with AF have yielded insightful results. In analyzing a wealth of data spanning various studies, the review found a landscape of outcomes associated with standard and reduced dosing strategies. Standard dosing demonstrated a consistent and statistically significant reduction in the risk of composite effectiveness outcomes compared to reduced dosing (HR: 0.89; 95% CI: 0.77-1.02, P=0.10). Additionally, standard dosing exhibited a more favorable association with the prevention of ischemic stroke when compared to reduced dosing. Standard dosing demonstrated a lower risk of major bleeding events when compared to reduced dosing as a group (HR 0.80, 95% CI 0.65-0.99). A study by Patel et al. presented similar conclusions regarding the efficacy of standard-dose rivaroxaban, demonstrating a significant reduction in composite effectiveness outcomes compared to reduced dosing, consistent with our meta-analysis [3]. Contrastingly, a meta-analysis by Lee et al. reported outcomes that diverged slightly from our findings. While supporting the superior efficacy of standard dosing, the study suggested a more modest difference in safety outcomes between standard and reduced dosing regimens. These discrepancies may arise from variations in patient populations, study designs, or inclusion criteria, underscoring the importance of considering nuanced differences when interpreting results [37].

Another trial by Hylek et al. explored the safety profiles of DOACs in AF. Although not specific to rivaroxaban, the study highlighted the need for personalized anticoagulation strategies, considering individual patient characteristics and bleeding risk [38]. In a different research, Graham et al. and Fralick et al., the group comprised individuals prescribed lower doses (constituting 23% of the participants) with underlying health conditions that suggested a heightened vulnerability to variations in the effectiveness and safety of anticoagulants. While the occurrence of significant ischemic or hemorrhagic events was higher among patients taking rivaroxaban at either dose, the extent of both the relative and absolute risk escalation was most notable for those administered reduced doses. This emphasizes the critical significance of selecting the appropriate anticoagulant in this specific population [39,40]. A study by Stærk et al. revealed that by comparing standard doses, rivaroxaban exhibited a one-year standardized absolute risk for major bleeding of 2.78%, with corresponding absolute risk differences lower for dabigatran (−0.93%) and apixaban (−0.54%). Similar results were observed for major bleeding with reduced non-vitamin K antagonist oral anticoagulants (NOAC) doses [29].

The previously discussed American studies by Graham et al., Hernandez and Zhang, Noseworthy et al., and another meta-analysis by Bai et al. also found an increased risk of bleeding associated with rivaroxaban compared with dabigatran [41-44]. Huang et al., conducted in Taiwan, evaluated rivaroxaban's effectiveness in preventing ischemic stroke among Asians with non-valvular AF. Results showed that rivaroxaban, particularly at 20 mg and 15 mg doses, significantly lowered the risk of VTE and ICH compared to warfarin. Both 20 mg and 15 mg doses were associated with a reduced risk of ischemic stroke, emphasizing their efficacy. However, the 10 mg dosage did not show the same risk reduction for ischemic stroke [18].

A recent study by Jansson et al. showed the comparison of reduced-dose DOACs to high time in therapeutic range (TTR) warfarin treatment. It revealed a clinically significant and favorable effectiveness and safety profile for reduced-dose DOACs. These are notably linked to a substantially reduced risk of intracranial bleeding. When contrasted with a warfarin-treated cohort, treatment with reduced-dose DOACs is associated with a lower risk of major bleeding and all-cause stroke. Specifically, rivaroxaban treatment demonstrates more effective prevention of ischemic stroke but comes with a higher risk of major bleeding [19].

Efficacy

Concerning effectiveness, we noted a dose-dependent trend, with an increasing rivaroxaban dose (from 10 mg to 15 mg and 20 mg) correlating with a significantly lower risk of ischemic stroke compared to warfarin. The risk reduction was particularly pronounced in the 20 mg group, suggesting that the standard dosing regimen (20 mg daily) may be more suitable, especially in patients without heightened bleeding risk. In contrast to the ROCKET AF and J-ROCKET AF studies, where the risk of ischemic stroke was comparable between rivaroxaban and warfarin, in many studies Huang et al. patients exhibited a lower baseline risk, possibly explaining rivaroxaban's heightened effectiveness [18,45,46]. Additionally, the rivaroxaban group in Bauersachs et al. showed a significantly lower risk of venous thromboembolism (VTE), indicating its efficacy in preventing VTE among AF patients [47].

Safety

Regarding safety, we observed a notably lower risk of ICH and a similar risk of gastrointestinal bleeding (GIB) in patients undergoing rivaroxaban treatment. Surprisingly, our data indicated that the 20 mg dose was paradoxically linked to a decreased risk of GIB. Conversely, the 10 mg dosage did not significantly decrease the risk of ICH. These outcomes may be attributed to between-group variations. In our study, physicians determined the dosage based on clinical judgment, leading to the 20 mg group having potentially more robust patients with lower bleeding risk, while the 10 mg group comprised more fragile individuals with higher bleeding risk, explaining the less pronounced reduction in ICH risk. However, Gozzo et al. show a high frequency of low-dose prescriptions of NOACs in patients with AF [48]. Older age, renal disease, bleeding risk, and the concomitant use of drugs predisposing to bleeding determined the choice of reduced dose. In Di Lullo et al., CKD patients, rivaroxaban was not associated with cerebrovascular events and/or major bleeding episodes in the first months of therapy [49].

The current study reaffirms that standard dosing of rivaroxaban consistently demonstrates superior efficacy in preventing composite effectiveness outcomes and ischemic strokes compared to reduced dosing. This has clear implications for AF patients with varying thromboembolic risk profiles. Clinicians may lean toward standard dosing, particularly in individuals with a higher risk of stroke, ensuring robust protection against ischemic events. Understanding the nuanced differences in safety outcomes is crucial in the clinical decision-making process. The lower risk of major bleeding, GIB, and intracranial bleeding associated with standard dosing implies that, in certain patient populations, the benefits of standard dosing may outweigh the potential risks. Conversely, reduced dosing may be considered for patients at higher bleeding risk, necessitating a judicious approach tailored to individual patient characteristics. By emphasizing the effectiveness of standard dosing in routine care settings, clinicians are encouraged to align their practices with established evidence. The translation of evidence into practice ensures that AF patients receive anticoagulation therapy that is not only evidence-based but also reflective of real-world effectiveness and safety.

While this analysis advances our understanding of rivaroxaban dosing in AF, several avenues for future research emerge. First, investigations into subpopulations, such as elderly patients or those with specific comorbidities, could provide tailored insights into the most effective and safe dosing strategies for these groups. Exploring the impact of rivaroxaban dosing on patient-reported outcomes and quality of life represents an essential yet underexplored dimension. Long-term, real-world studies are warranted to monitor the persistence of treatment effects and assess the durability of outcomes over extended periods.

Strengths

The comprehensive search strategy employed ensured the inclusion of a diverse range of studies, providing a broad representation of the current literature on rivaroxaban dosing in AF. Rigorous inclusion criteria were applied, bolstering the quality of the included studies and minimizing the ROB. Additionally, the use of a meta-analytic approach allowed for a quantitative synthesis of data, enabling a more robust assessment of the efficacy and safety outcomes associated with different dosing regimens. The incorporation of recent literature, focusing on studies published from 2017 onward, ensures the currency of the analysis and relevance to contemporary clinical practice. These methodological strengths collectively contribute to the robustness of the study's conclusions, offering valuable insights into the optimal use of rivaroxaban in AF.

Limitations 

Although the study investigated the right outcomes and measures for analysis and assessment, it had several limitations. First, the sample sizes taken for meta-analysis could not be standardized according to usual protocols. We used study characteristics in consideration but did not consider methodological characteristics of studies. Second, very few primary studies were utilized to assess the effectiveness (outcome domain) for such a large sample size. Third, we evaluated the overall combined effect of all sample sizes, but within-group and sub-group analyses were not performed. Several studies have demonstrated that the results of the final analysis can be significantly altered when population demographics are sub-grouped into effect sizes.

## Conclusions

The clinical implications drawn from this analysis advocate for a thoughtful and patient-centered approach to anticoagulation therapy in AF. There is little impact of difference in the dosing of rivaroxaban with slightly reduced risk of stroke risk with standard dosing but with raised bleeding events. By considering the individualized needs of patients, clinicians can navigate the complexities of stroke prevention and bleeding risk, optimizing outcomes in this high-risk population.

## References

[1] Hindricks G, Potpara T, Dagres N (2021). 2020 ESC guidelines for the diagnosis and management of atrial fibrillation developed in collaboration with the European Association for Cardio-Thoracic Surgery (EACTS): the task force for the diagnosis and management of atrial fibrillation of the European Society of Cardiology (ESC) developed with the special contribution of the European Heart Rhythm Association (EHRA) of the ESC. Eur Heart J.

[2] Kornej J, Börschel CS, Benjamin EJ, Schnabel RB (2020). Epidemiology of atrial fibrillation in the 21st century: novel methods and new insights. Circ Res.

[3] Patel MR, Mahaffey KW, Garg J (2011). Rivaroxaban versus Warfarin in nonvalvular atrial fibrillation. N Engl J Med.

[4] Eikelboom JW, Connolly SJ, Bosch J (2017). Rivaroxaban with or without aspirin in stable cardiovascular disease. N Engl J Med.

[5] Connolly SJ, Ezekowitz MD, Yusuf S (2009). Dabigatran versus warfarin in patients with atrial fibrillation. N Engl J Med.

[6] Kirchhof P, Benussi S, Kotecha D (2016). 2016 ESC Guidelines for the management of atrial fibrillation developed in collaboration with EACTS. Eur J Cardiothorac Surg.

[7] Owolabi MO, Thrift AG, Mahal A (2022). Primary stroke prevention worldwide: translating evidence into action. Lancet Public Health.

[8] Singh R, Emmady PD (2023). Rivaroxaban.

[9] Ashton V, Mudarris L, Moore KT (2021). The pharmacology, efficacy, and safety of rivaroxaban in obese patient populations. Am J Cardiovasc Drugs.

[10] Ouzzani M, Hammady H, Fedorowicz Z, Elmagarmid A (2016). Rayyan-a web and mobile app for systematic reviews. Syst Rev.

[11] Page MJ, McKenzie JE, Bossuyt PM (2021). The PRISMA 2020 statement: an updated guideline for reporting systematic reviews. BMJ.

[12] Chalmers TC, Frank CS, Reitman D (1990). Minimizing the three stages of publication bias. J Am Med Assoc.

[13] Higgins JP, Altman DG, Gøtzsche PC (2011). The Cochrane Collaboration’s tool for assessing risk of bias in randomised trials. BMJ.

[14] (2020). Review manager (RevMan) [computer program]: version 5.4. https://training.cochrane.org/system/files/uploads/protected_file/RevMan5.4_user_guide.pdf.

[15] Ray WA, Chung CP, Stein CM (2021). Association of rivaroxaban vs apixaban with major ischemic or hemorrhagic events in patients with atrial fibrillation. J Am Med Assoc.

[16] Lau WC, Torre CO, Man KK (2022). Comparative effectiveness and safety between apixaban, dabigatran, edoxaban, and rivaroxaban among patients with atrial fibrillation: a multinational population-based cohort study. Ann Intern Med.

[17] Alberts M, Chen YW, Lin JH, Kogan E, Twyman K, Milentijevic D (2020). Risks of stroke and mortality in atrial fibrillation patients treated with rivaroxaban and warfarin. Stroke.

[18] Huang HY, Lin SY, Cheng SH, Wang CC (2018). Effectiveness and safety of different rivaroxaban dosage regimens in patients with non-valvular atrial fibrillation: a nationwide, population-based cohort study. Sci Rep.

[19] Jansson M, Själander S, Sjögren V, Björck F, Renlund H, Norrving B, Själander A (2023). Reduced dose direct oral anticoagulants compared with warfarin with high time in therapeutic range in nonvalvular atrial fibrillation. J Thromb Thrombolysis.

[20] Carnicelli AP, Hong H, Connolly SJ (2022). Direct oral anticoagulants versus Warfarin in patients with atrial fibrillation: patient-level network meta-analyses of randomized clinical trials with interaction testing by age and sex. Circ.

[21] Guimarães HP, Lopes RD, de Barros E Silva PG (2020). Rivaroxaban in patients with atrial fibrillation and a bioprosthetic mitral valve. N Engl J Med.

[22] Perera KS, Ng KK, Nayar S (2020). Association between low-dose rivaroxaban with or without aspirin and ischemic stroke subtypes: a secondary analysis of the compass trial. JAMA Neurol.

[23] Blumer V, Rivera M, Corbalán R (2021). Rivaroxaban versus warfarin in patients with atrial fibrillation enrolled in Latin America: insights from ROCKET AF. Am Heart J.

[24] Akao M, Yasuda S, Kaikita K (2021). Rivaroxaban monotherapy versus combination therapy according to patient risk of stroke and bleeding in atrial fibrillation and stable coronary disease: AFIRE trial subanalysis. Am Heart J.

[25] Mehra MR, Vaduganathan M, Fu M (2019). A comprehensive analysis of the effects of rivaroxaban on stroke or transient ischaemic attack in patients with heart failure, coronary artery disease, and sinus rhythm: the COMMANDER HF trial. Eur Heart J.

[26] Healey JS, Gladstone DJ, Swaminathan B (2019). Recurrent stroke with rivaroxaban compared with aspirin according to predictors of atrial fibrillation: secondary analysis of the NAVIGATE ESUS randomized clinical trial. JAMA Neurol.

[27] Zhang L, Yan X, Fox KA (2020). Associations between model-predicted rivaroxaban exposure and patient characteristics and efficacy and safety outcomes in patients with non-valvular atrial fibrillation. J Thromb Thrombolysis.

[28] Shrestha S, Baser O, Kwong WJ (2018). Effect of renal function on dosing of non-vitamin K antagonist direct oral anticoagulants among patients with nonvalvular atrial fibrillation. Ann Pharmacother.

[29] Staerk L, Gerds TA, Lip GY (2018). Standard and reduced doses of dabigatran, rivaroxaban and apixaban for stroke prevention in atrial fibrillation: a nationwide cohort study. J Intern Med.

[30] Pisters R, van Vugt SP, Brouwer MA (2017). Real-life use of rivaroxaban in the Netherlands: data from the Xarelto for prevention of stroke in patients with atrial fibrillation (XANTUS) registry. Neth Heart J.

[31] Ntaios G, Pearce LA, Veltkamp R (2020). Potential embolic sources and outcomes in embolic stroke of undetermined source in the NAVIGATE-ESUS trial. Stroke.

[32] Nagao T, Suzuki H, Matsunaga S (2019). Impact of periprocedural anticoagulation therapy on the incidence of silent stroke after atrial fibrillation ablation in patients receiving direct oral anticoagulants: uninterrupted vs. interrupted by one dose strategy. Europace.

[33] Guimarães HP, de Barros E Silva PG, Liporace IL (2021). A randomized clinical trial to evaluate the efficacy and safety of rivaroxaban in patients with bioprosthetic mitral valve and atrial fibrillation or flutter: rationale and design of the RIVER trial. Am Heart J.

[34] Karthikeyan G, Connolly SJ, Ntsekhe M (2020). The INVICTUS rheumatic heart disease research program: rationale, design and baseline characteristics of a randomized trial of rivaroxaban compared to vitamin K antagonists in rheumatic valvular disease and atrial fibrillation. Am Heart J.

[35] Providência R, Grove EL, Husted S, Barra S, Boveda S, Morais J (2014). A meta-analysis of phase III randomized controlled trials with novel oral anticoagulants in atrial fibrillation: comparisons between direct thrombin inhibitors vs. factor Xa inhibitors and different dosing regimens. Thromb Res.

[36] Miyazaki M, Matsuo K, Uchiyama M (2020). Inappropriate direct oral anticoagulant dosing in atrial fibrillation patients is associated with prescriptions for outpatients rather than inpatients: a single-center retrospective cohort study. J Pharm Health Care Sci.

[37] Lee HF, Chan YH, Tu HT (2018). The effectiveness and safety of low-dose rivaroxaban in Asians with non-valvular atrial fibrillation. Int J Cardiol.

[38] Hylek EM, Held C, Alexander JH (2014). Major bleeding in patients with atrial fibrillation receiving apixaban or warfarin: the ARISTOTLE trial (apixaban for reduction in stroke and other thromboembolic events in atrial fibrillation): predictors, characteristics, and clinical outcomes. J Am Coll Cardiol.

[39] Graham DJ, Baro E, Zhang R (2019). Comparative stroke, bleeding, and mortality risks in older medicare patients treated with oral anticoagulants for nonvalvular atrial fibrillation. Am J Med.

[40] Fralick M, Colacci M, Schneeweiss S, Huybrechts KF, Lin KJ, Gagne JJ (2020). Effectiveness and safety of apixaban compared with rivaroxaban for patients with atrial fibrillation in routine practice: a cohort study. Ann Intern Med.

[41] Graham DJ, Reichman ME, Wernecke M (2016). Stroke, bleeding, and mortality risks in elderly Medicare beneficiaries treated with dabigatran or rivaroxaban for nonvalvular atrial fibrillation. JAMA Intern Med.

[42] Hernandez I, Zhang Y (2017). Stroke, bleeding, and mortality risks in elderly medicare beneficiaries treated with dabigatran or rivaroxaban for nonvalvular atrial fibrillation. Am J Cardiovasc Drugs.

[43] Noseworthy PA, Yao X, Abraham NS, Sangaralingham LR, McBane RD, Shah ND (2016). Direct comparison of dabigatran, rivaroxaban, and apixaban for effectiveness and safety in nonvalvular atrial fibrillation. Chest.

[44] Bai Y, Deng H, Shantsila A, Lip GY (2017). Rivaroxaban versus dabigatran or warfarin in real-world studies of stroke prevention in atrial fibrillation: systematic review and meta-analysis. Stroke.

[45] Patel MR, Mahaffey KW, Garg J (2011). Rivaroxaban versus warfarin in nonvalvular atrial fibrillation. N Engl J Med.

[46] Hori M, Matsumoto M, Tanahashi N (2012). Rivaroxaban vs. warfarin in Japanese patients with atrial fibrillation: the J-ROCKET AF study. Circ J.

[47] Bauersachs R, Berkowitz SD, Brenner B (2010). Oral rivaroxaban for symptomatic venous thromboembolism. N Engl J Med.

[48] Gozzo L, Di Lenarda A, Mammarella F (2021). Starting dose and dose adjustment of non-vitamin K antagonist oral anticoagulation agents in a nationwide cohort of patients with atrial fibrillation. Sci Rep.

[49] Di Lullo L, Tripepi G, Ronco C (2018). Safety and effectiveness of rivaroxaban and warfarin in moderate-to-advanced CKD: real world data. J Nephrol.

